# Supplementary material for: Characterisation of Antennal Sensilla and Electroantennography Responses of the Dung Beetles Bubas bison, Onitis aygulus and Geotrupes spiniger (Coleoptera: Scarabaeoidea) to Dung Volatile Organic Compounds
Source: Insects. 2023 Jul 12;14(7):627. doi: 10.3390/insects14070627 (PMC10380661; doi:10.3390/insects14070627)
Supplement: Supplementary file 1 [file insects-14-00627-s001.zip › insects-2471043-supplementary.pdf]

Supplementary information

Table S1 Chemical compounds used for EAG study.

Chemical	CAS number	Purity %
phenol	108-95-2	99.0-100.5
skatole	83-34-1	98
indole	120-72-9	≥ 99
butanone	78-93-3	≥ 99.0
butyric acid	107-92-6	≥ 99
p-cresol	106-44-5	99
eucalyptol	470-82-6	99
toluene	108-88-3	99.9
dimethyl sulfide	75-18-3	≥ 99.0
dimethyl disulfide	624-92-0	≥ 99.0

Table S2: Lengths of antennal segments (mean ± SE) of adult beetles compared between sexes (*n*=3, **p* <0.05)

Antennal segment	Length (µm) (Mean ±SE)					
	Bubas bison		*Onitis aygulus*		*Geotrupes spiniger*	
	Male	Female	Male	Female	Male	Female
Scape	676.23 ± 72.86	729.02 ± 52.89	1264.87 ± 126.11	951.21 ± 31.78	920.92 ± 75.062	953.56 ± 9.02
Pedicel	178.71 ± 4.37	166.63 ± 9.03	213.06 ± 4.65	217.71 ± 20.61	269.95 ± 17.10	260.96 ± 11.70
Flagellomere 1	172.97 ± 2.69	185.46 ± 14.73	200.17 ± 5.83*	169.73 ± 5.14	311.43 ± 36.67	325.40 ± 10.53
Flagellomere 2	148.39 ± 4.71	140.84 ± 7.93	148.66 ± 1.40 *	110.14 ± 8.70	206.75 ± 8.32	210.40 ± 16.14
Flagellomere 3	111.41 ± 6.94	102.10 ± 2.61	94.32 ± 4.99	74.68 ± 13.83	284.82 ± 30.37	265.31 ± 19.63
Flagellomere 4	70.23 ± 10.40	51.87 ± 8.96	45.81 ± 1.89	58.38 ± 5.59	178.85 ± 18.25	163.06 ± 14.80
Flagellomere 5	-	-	-	-	111.02 ± 10.08	91.73 ± 18.37
Flagellomere 6	-	-	-	-	60.60 ± 3.84	55.29 ± 12.85
Funicle	502.99 ± 15.58	480.29 ± 18.36	488.97 ± 11.43	412.93 ± 32.57	1153.48 ± 95.96	1111.17 ± 89.32
L1	993.31 ± 48.72	1023.61 ± 64.11	1502.41 ± 104.75	1284.00 ± 54.40	1173.78 ± 41.00	1201.09 ± 41.83
L2	821.40 ± 4.23	899.55 ± 61.67	1142.27 ± 63.69	990.62 ± 63.95	917.07 ± 40.95	920.46 ± 30.55
L3	740.60 ± 29.50	834.61 ± 65.99	1164.41 ± 64.28	908.24 ± 86.73	803.87 ± 35.63	902.92 ± 21.86

Table S3: Pooled sensilla density data for antennal sensilla, ST and SB (Mean ± SE) in different surfaces. No significant differences were found among sensilla surfaces

Species	Antennal section	Sensilla density/ 100µm² (Mean ±SE)	
		**Male**	**Female**
Bubas bison	L1 proximal	0.33 ± 0.25	0.23 ± 0.25
	L1 distal	1.30 ± 0.25	1.28 ± 0.25
	L2 proximal	0.57 ± 0.31	0.67 ± 0.25
	L2 distal	1.26 ± 0.25	0.25 ± 0.31
	L3 proximal	0.69 ± 0.25	0.44 ± 0.31
	L3 distal	0.79 ± 0.25	0.81 ± 0.25
Onitis aygulus	L1 proximal	0.13 ± 0.12	0.19 ± 0.11
	L1 distal	0.87 ± 0.14	0.86 ± 0.11
	L2 proximal	0.87 ± 0.12	0.86 ± 0.13
	L2 distal	0.85 ± 0.14	0.95 ± 0.11
	L3 proximal	0.97 ± 0.12	0.94 ± 0.11
	L3 distal	0.86 ± 0.12	0.92 ± 0.13
Geotrupes spiniger	L1 proximal	0.51 ± 0.09	0.47 ± 0.06
	L1 distal	1.29 ± 0.41	1.16 ± 0.34
	L2 proximal	0.64 ± 0.03	0.56 ± 0.08
	L2 distal	1.31 ± 0.39	1.19 ± 0.33
	L3 proximal	0.58 ± 0.06	0.60 ± 0.09
	L3 distal	0.74 ± 0.08	0.74 ± 0.06

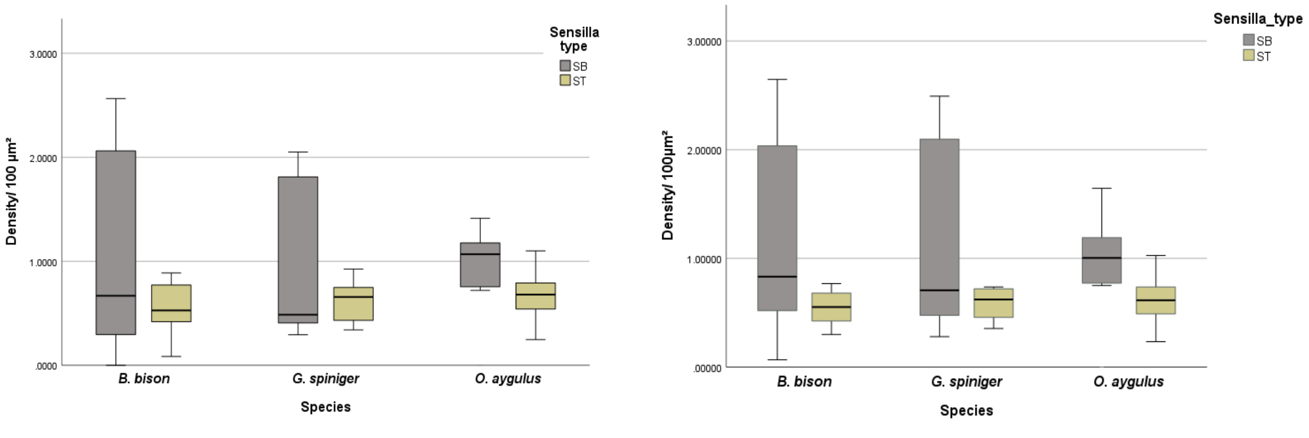

Male

Figure S1 Overall distribution of ST and SB in three species

Table S4: EAG responses (mean ± SE) of adult female *Bubas bison*, *Onitis aygulus* and *Geotrupes spiniger* to dung VOCs compared to the control. Significance from *t* test $^{*}p < 0.05$, $^{**}p < 0.01$, $^{***}p < 0.001$, $^{****}p < 0.0001$

	Bubas bison		*Onitis aygulus*		*Geotrupes spiniger*	
	compound	control	compound	control	compound	control
mix	0.489 ± 0.085**	0.103 ± 0.016	0.311 ± 0.037**	0.053 ± 0.017	0.440 ± 0.046**	0.096 ± 0.013
skatole	0.556 ± 0.122**	0.124 ± 0.068	0.213 ± 0.055*	0.038 ± 0.018	0.442 ± 0.043***	0.071 ± 0.007
indole	0.387 ± 0.201	0.083 ± 0.036	0.296 ± 0.126	0.089 ± 0.047	0.426 ± 0.100*	0.075 ± 0.020
eucalyptol	0.393 ± 0.166	0.056 ± 0.018	0.107 ± 0.018*	0.038 ± 0.006	0.221 ± 0.034**	0.044 ± 0.006
butyric acid	0.354 ± 0.051*	0.092 ± 0.018	0.178 ± 0.039*	0.050 ± 0.012	0.302 ± 0.050**	0.061 ± 0.013
DMS	0.436 ± 0.075*	0.066 ± 0.017	0.196 ±0.046	0.010 ± 0.010	0.211 ± 0.047*	0.051 ± 0.013
DMDS	0.269 ± 0.089	0.048 ± 0.021	0.120 ± 0.022	0.028 ± 0.014	0.269 ± 0.047**	0.051 ± 0.013
butanone	0.292 ± 0.117	0.038 ± 0.010	0.246 ± 0.062	0.067 ± 0.014	0.231 ± 0.036**	0.060 ± 0.008
toluene	0.402 ± 0.069**	0.063 ± 0.012	0.396 ± 0.057*	0.024 ± 0.013	0.218 ± 0.020***	0.060 ± 0.009
p-cresol	0.498 ± 0.136*	0.072 ± 0.024	0.289 ±0.079*	0.048 ± 0.015	0.660 ± 0.049****	0.091 ± 0.020
phenol	0.319 ± 0.063*	0.082 ± 0.037	0.328 ± 0.064*	0.086 ± 0.022	0.357 ± 0.072*	0.069 ± 0.010

Table S5: Relative EAG responses (mean ± SE) for each test compound compared among adult female *B. bison*, *O. aygulus* and *G. spiniger* to dung VOCs. Different lowercase letters among species in skatole and *p*-cresol are significantly different at $p < 0.05$, as determined by ANOVA followed by LSD. $^{*}p < 0.05$

Compound	Relative EAG response (Mean ±SE)			*F*	*p*
	Bubas bison	*Onitis aygulus*	*Geotrupes spiniger*		
mix	0.489 ± 0.063	0.311 ± 0.082	0.440 ± 0.082	1.49	0.252
skatole	0.530 ± 0.079*, a	0.194 ± 0.087b	0.418 ± 0.079ab	5.52	0.029
indole	0.361 ± 0.148	0.273 ± 0.170	0.391 ± 0.132	0.15	0.861
eucalyptol	0.355 ± 0.104	0.089 ± 0.135	0.198 ± 0.095	1.33	0.305
butyric acid	0.334 ± 0.051	0.155 ± 0.059	0.280 ± 0.059	2.73	0.113
DMS	0.408 ± 0.060	0.190 ±0.096	0.196 ± 0.055	3.85	0.058
DMDS	0.245 ± 0.056	0.111 ± 0.056	0.251 ± 0.044	2.2	0.173
butanone	0.283 ± 0.078	0.227 ± 0.090	0.214 ± 0.064	0.24	0.789
toluene	0.372 ± 0.055	0.381 ± 0.084	0.194 ± 0.059	2.95	0.088
p-cresol	0.449 ± 0.091ab	0.272 ± 0.108b	0.630 ± 0.108*, a	7.73	0.011
phenol	0.305 ± 0.065	0.296 ± 0.084	0.333 ± 0.065	0.07	0.929
